# Case report: cerebral thromboembolism in an unconscious COVID-19 patient in intensive care

**DOI:** 10.11604/pamj.2021.38.373.29034

**Published:** 2021-04-15

**Authors:** Edhem Unver, Ufuk Kuyrukluyildiz, Erdal Karavas, Hakan Gokalp Tas

**Affiliations:** 1 Department of Chest Disease, Faculty of Medicine, Erzincan Binali Yildirim University, Erzincan, Turkey,; 2 Department of Anesthesiology and Reanimation, Faculty of Medicine, Erzincan Binali Yildirim University, Erzincan, Turkey,; 3 Department of Radiology, Faculty of Medicine, Erzincan Binali Yildirim University, Erzincan, Turkey,; 4 Department of Anesthesiology and Reanimation, Siran Government Hospital, Gumushane, Turkey

**Keywords:** COVID-19, pneumonia, tromboembolia, case report, intensive care

## Abstract

Although the severity of coronavirus disease 2019 (COVID-19) being more frequently related to acute respiratory distress syndrome and acute cardiac and renal injuries, thromboembolic events have been increasingly reported. Acute respiratory distress syndrome due to SARS-CoV-2 (Severe Acute Respiratory Syndrome - Corona Virus 2) often requires intensive care follow-up. As well as respiratory failure, the SARS-CoV-2 may cause central nervous system (CNS) involvement. The pandemic has raised many challenges in managing critically ill older adults, a population preferentially killed by COVID-19. The mortality and morbidity rates are extremely high in critically ill patients with COVID-19. Recent studies have reported the potential development of a hypercoagulable state in COVID-19. Viral infections and hypoxia may cause these state. It is increasingly reported that thromboembolic events are associated with a poor prognosis. Due to these thromboembolic complications, COVID-19 patients often have neurological symptoms. These symptoms may not be observed in intensive care patients who are sedated. We report one case who was sedated COVID-19 pneumonia and who was later diagnosed with cerebral venous thrombosis with cranial imaging when he could not awaken even though sedation was discontinued. Since COVID-19 causes intense thrombotic susceptibility due to cytokine storm, cerebrovascular thromboembolic complications associated with COVID-19 infection should be considered first and foremost for unconsciousness ventilated patients. Severe and potentially cerebral thrombosis may prolong the patient´s stay in intensive care.

## Introduction

Severe Acute Respiratory Syndrome - Corona Virus 2 (SARS-CoV-2) was detected from a nasopharyngeal swab in a patient with atypical pneumonia in Wuhan, China, by the Chinese Center for Disease Control and Prevention on January 7, 2020 [1]. Patients infected with SARS-CoV-2, also known as coronavirus disease 2019 (COVID-19), mainly develop respiratory and digestive symptoms [2]. It has been suggested early in the epidemics that SARS-CoV-2 may also invade the central nervous system and be responsible for neurological signs [3]. The mortality and morbidity rates are extremely high in critically ill patients with COVID-19. Although the severity of COVID-19 is more frequently associated with acute respiratory distress syndrome and acute cardiac and renal injury, it is increasingly reported that thromboembolic events are associated with a poor prognosis and these events may develop independently of pulmonary or respiratory symptoms at presentation [4]. Recent studies have reported the potential development of the hypercoagulable state in COVID-19 [5]. Viral infections can increase the dysfunction of endothelial cells, leading to excessive thrombin formation and inhibition of fibrinolysis [6]. In addition, hypoxemia is linked to the activation of hypoxia-related genes that mediate coagulation and fibrinolysis with an increase in blood viscosity and promotes thrombotic events [7]. Due to these thromboembolic complications, COVID-19 patients often have neurological symptoms [8]. In particular, cerebral venous thrombosis can manifest itself with a wide variety of neurological signs and symptoms [9]. We would like to report a case that developed pneumonia due to COVID-19 infection and was unconscious after being intubated and followed up on mechanical ventilator, who was later diagnosed with cerebral venous thrombosis with cranial imaging and continued to be followed up in the intensive care unit.

## Patient and observation

**Figure 1:**
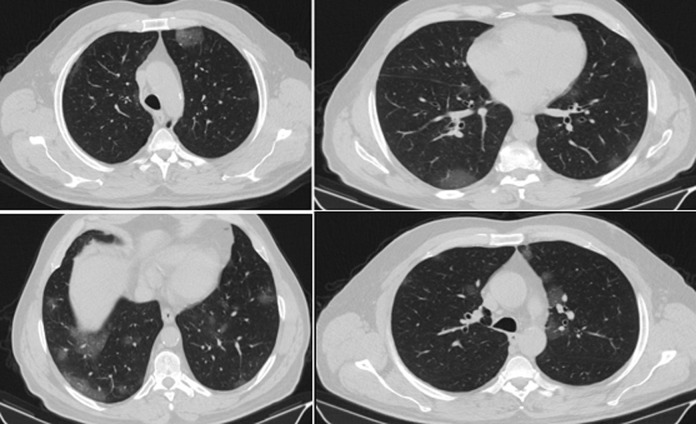
chest computed tomography; widespread, predominantly peripheral multifocal ground glass densities were observed in both lungs

A 64-year-old healthy male patient was admitted to the emergency clinic of Erzincan Binali Yildirim University Mengucek Gazi Training and Research Hospital after 10 days of worsening cough and sore throat symptoms. He had no additional respiratory pathology. On physical examination; the patient was conscious, cooperative, and lung and heart auscultation was normal. Oxygen saturation with pulse oximetry was 91%. In blood tests; Platelet count was 136000, CRP was 72, lymphocyte was low, D-dimer and CK were high, coagulation tests were normal. The patient was evaluated in terms of COVID-19 due to low oxygen saturation along with lymphopenia and high CRP. He was hospitalized for bilateral ground glass opacities on his thorax CT (Figure 1). Real time PCR result was positive. The patient was hospitalized with COVID-19 pre-diagnosis. Azithromycin 500 mg, Hydroxychloroquine 2*200 mg, Oseltamivir 2*75mg, low molecular weight heparin (enoxaparin) 2*6000 IU for 5 days and proton pump inhibitor for gastric ulcer prophylaxis treatment was initiated.

CRP, Procalsitonin, D-Dimer, Ferritin, CK results gradually increased on the 3^rd^ day of hospitalization. When nasal oxygen therapy was reduced to less than 3 L/min, transcutaneous oxygen saturation fell below 90%. On lung auscultation, diffuse rales were detected in both lung bases. Since this situation showed that the severity of COVID-19 pneumonia increased despite the treatment, the patient was taken to the intensive care unit (ICU).65 LT/min high flow oxygen therapy was applied for 2 days in intensive care unit. At the end of the second day, although he received high flow oxygen therapy, the general condition of the patient deteriorated and respiratory distress increased. Therefore, although the patient was oriented and cooperated, he was intubated electively with the diagnosis of acute respiratory distress syndrome (ARDS). The patient was sedated with midazolam and lung protective ventilation treatment was initiated with low tidal volume and optimally high PEEP. Favipiravir was added to the treatment and hydroxychloroquine treatment was extended for 5 days. 6000 IU Enoxaparine was applied twice a day for thromboembolism prophylaxis. In addition to sedation with midazolam, rocuronium was added as a muscle relaxant. Tocilizumab and methylprednisolone 60 mg were added to the treatment of the patients who had WBC: 3100, plt 101000, Lymphocyte: 300 (9.6%), CRP: 489, PCT: 3.1, Ferritin > 1650, D Dimer > 100.000 in blood tests on the 6^th^ day of the ICU follow-up.

**Figure 2:**
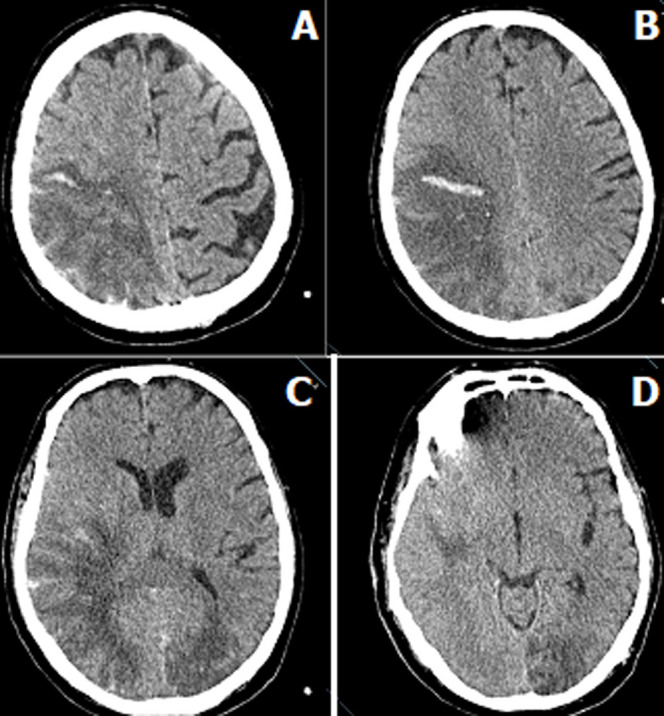
cranial computed tomography; A) hypodense areas in the frontal, parietal and temporal lobes on the right, B) occipital lobes, erasing of the cortical sulci due to edema in these areas, C) compression of the posterior horn of the right ventricle, D) hyperdense foci compatible with bilateral hemorrhagic changes accompanying the hypodense areas

With the current treatment, we planned extubation by discontinuing sedation on the 16^th^ day in the intensive care unit, due to the improvement of the patient´s clinical condition, decrease in fever, regression of radiological findings, improvement in D-Dimer, Ferritin, CRP, lymphocyte count, and Fibrinogen parameters, which are bad prognostic factors. The patient was not fully conscious 72 hours after sedation was stopped. The Glaskow Coma Scale score was 6. Therefore, it was decided to have a cranial CT scan of the patient. In cranial CT angiography, occlusion in the vessels feeding some regions that appear compatible with ischemia in vascular structures and also bleeding areas in these regions were detected (Figure 2). Following these findings, the patient's intensive care follow-up continued with the diagnosis of cerebrovascular occlusion secondary to COVID-19 pneumonia, and the patient died on the 45^th^day of hospitalization.

## Discussion

There may be a hypothesis that viral-mediated degradation of endothelin in patients infected with SARS-CoV-2 plays a key role in thrombus formation. Related to this, viral particles, inflammatory cell accumulation and apoptosis have been detected in the systemic vascular endothelium [10, 11]. It is now known that COVID-19 infection causes an intense inflammatory response in the body. This cytokine storm due to COVID-19 can cause damage especially in the brain. As a result, acute brain diseases can be observed. According to Mao *et al*. 36.4% of COVID-19-infected cases had neurological symptoms. Acute cerebrovascular disease was reported in 5.7% of all COVID-19 cases [8]. It is very important to decide the intensity of thromboprophylaxis, especially in patients with high thrombotic risk in intensive care [12]. An anticoagulant drug such as low molecular weight heparin and / or dipyridamole should be administered to prevent possible thromboembolic complications. However, cytokine storm in COVID-19 patients causes thrombotic predisposition and standard therapeutic doses of thrombolytics are insufficient in some cases. Consciousness should be followed before sedation. When the decision of extubation is made in patients who require mechanical ventilation under sedation due to respiratory failure, consciousness should be especially evaluated [13]. In this case, enoxaparin was administered to prevent thromboembolic complications from the moment the patient was admitted to the intensive care unit with the diagnosis of COVID-19.The patient was conscious and cooperative until he was intubated due to respiratory failure. COVID-19 pneumonia treated after intubation. Since the patient was not conscious after the treatment, the presence of cerebrovascular occlusion was detected as a result of the examinations and radiological imaging.

## Conclusion

In conclusion, the patients who were ventilated mechanically due to COVID-19 infection and whose consciousness was not restored even though sedation was discontinued, cerebrovascular thromboembolic complications associated with COVID-19 infection should be considered first and foremost, even if anticoagulant agents are present in the treatment protocol, since COVID-19 causes intense thrombotic tendency due to cytokine storm.
